# The Rational Use of Hormone Replacement Therapy in Older Adults

**DOI:** 10.1093/geroni/igab046.1812

**Published:** 2021-12-17

**Authors:** Jennifer Mammen, Eleanor Simonsick, Eleanor Simonsick

## Abstract

Multiple hormone levels are lower in older compared to younger adults, but the clinical implications are controversial. Lower thyroid hormone, testosterone, estrogen and vitamin D are variously associated with metabolic syndrome, sarcopenia, low bone density and cognitive decline, for which treatment has been recommended in the past. However, evidence from the T Trials of testosterone therapy failed to show benefit for endpoints other than sexual function. In the Women’s Health Initiative, a large RCT of estrogen replacement in older women, found evidence of an interaction between estrogen therapy and metabolic risk factors such as diabetes that actually exacerbates risk of cognitive decline. Longitudinal observation of thyroid hormone and thyrotropin patterns in the Baltimore Longitudinal Study of Aging demonstrate heterogeneity that might account for a lack of benefit in studies of treatment for subclinical hypothyroidism in older adults. At the same time, new data suggest the need for a more aggressive threshold for vitamin D in older adults, with a lower threshold associated with a drop in physical function compared to younger adults. Complexity in the regulation of hormonal pathways and the downstream effects on target tissues means multiple individuals with similar hormone levels may have different underlying physiology, with divergent clinical needs. Changes in activity and diet common during aging, and exacerbated by the pandemic, lead to physical and mood changes associated with hormonal dysfunction in popular culture and patient requests for evaluation. The ultimate goal should be personalized treatment decisions based on comprehensive evaluation and pathophysiology.

